# Fabrication of Paper Microfluidic Chips via Wax Soft Lithography

**DOI:** 10.3390/mi17050512

**Published:** 2026-04-23

**Authors:** Xinyi Chen, Jie Zhou, Jiahua Zhong, Zitong Ye, Qinghao He, Hao Chen, Weijin Guo

**Affiliations:** Department of Biomedical Engineering, Shantou University, Shantou 515063, China

**Keywords:** paper microfluidics, soft lithography, wax patterning, glucose, POCT

## Abstract

Paper-based microfluidic devices (μPADs) have attracted significant attention for point-of-care testing (POCT), environmental monitoring, and food safety due to their low cost, ease of use, and minimal instrument dependence. However, fabricating high-resolution and reproducible microchannels on paper remains challenging. Conventional methods such as wax printing, photolithography, and inkjet printing are limited by resolution or equipment cost. Here, we present a low-cost, high-resolution fabrication method for μPADs, termed wax soft lithography, which combines wax printing with soft lithography. Through this method, microchannels with a minimum width of 234 ± 62 μm were consistently produced, and complex patterns were successfully fabricated, demonstrating high precision and reproducibility. As a proof-of-concept demonstration of device functionality, the fabricated μPADs were used to detect glucose in spiked urine samples, showing a concentration-dependent colorimetric response. This method provides an effective route for rapid production of high-resolution μPADs in resource-limited settings. With further validation before practical applications, this method shows promise for future development in POCT.

## 1. Introduction

Microfluidic technology has been widely employed in applications such as point-of-care testing [1,2], drug development [3,4,5], and environmental monitoring [6], owing to its high manipulation precision, minimal sample consumption, and high level of integration. Among various microfluidic platforms, paper-based microfluidic devices (μPADs) have garnered particular attention due to their ultra-low cost, simple fabrication, and intrinsic capability for pump-free fluid transport driven by capillary action [7,8]. In recent years, numerous applications on μPADs for point-of-care testing (POCT) have been developed. Paper is typically composed of an interwoven network of natural cellulose or nitrocellulose fibers, containing abundant micrometer-scale pores [9]. When paper comes into contact with an aqueous liquid, the liquid spontaneously wicks through the porous structure, driven by capillary forces arising from surface tension and solid–liquid interfacial interactions [10]. Hydrophobic agents—such as wax [9] or polydimethylsiloxane (PDMS) [11]—have been used to form well-defined hydrophobic barriers that restrict liquid penetration. By precisely engineering the geometry and spatial arrangement of these hydrophobic patterns, both the flow path and the wicking velocity of aqueous liquids on μPADs can be effectively modulated [12].

Achieving precise and reproducible fabrication of microfluidic channels on paper substrates remains a significant challenge [13]. Conventional approaches for constructing μPADs, such as wax printing, form hydrophobic patterns by depositing wax onto paper and melting it to allow penetration into the porous matrix [14,15]. Wax printers can achieve a decent resolution, as demonstrated by Tenda et al., who produced microfluidic channels of 228 ± 30 μm on paper [16]. Nevertheless, the gradual discontinuation of wax printers has been a problem for many researchers [16,17]. Photolithography technology enables the fabrication of high-resolution patterned microfluidic channels on filter paper with a minimum width of 90 μm by inducing cross-linking reactions of photosensitive materials under ultraviolet (UV) light irradiation [18]. Yet, this approach requires expensive equipment, specialized photoresists, and sophisticated processing procedures, rendering it unfeasible for low-cost and large-scale manufacturing [19,20,21]. Inkjet printing precisely deposits functional liquids such as hydrophobic agents or solvents onto paper via an inkjet printer to create hydrophilic or hydrophobic patterns. Nevertheless, this process often requires multiple printing layers, which can lead to reduced resolution [22,23].

To address these issues, this study proposes an innovative and low-cost fabrication method for μPADs, namely wax soft lithography, which combines wax printing with soft lithography. Soft lithography has become one of the most widely used microfabrication techniques for microfluidic chips due to its high pattern fidelity and operational simplicity, relying on elastomeric materials to replicate and transfer microscale structures [24]. Among these, PDMS has become the primary material for soft lithography processes due to its excellent biocompatibility, gas permeability, and optical transparency [25]. Su et al. used laser cutting to prepare the template for molding PDMS and sealed the PDMS chip with a glass substrate, resulting in wax adhering to the glass surface after cooling before being transferred onto paper [26]. Their method achieved a minimum channel width of ∼654 μm. In this study, photolithography was used to fabricate the master mold, followed by PDMS replica molding to form the chip, which was sealed with a PDMS film to retain molten wax within the microchannels after cooling. Upon heating, the wax is transferred from the microchannels onto the paper substrate to create the predefined patterns. We systematically evaluated the minimum achievable microchannel width by fabricating channels of varying widths on paper using wax soft lithography. In addition, multiple pattern shapes were fabricated on paper. The resulting microchannels were successfully used to detect glucose of different concentrations in urine samples, which demonstrated the process reliability and application potential of the wax soft lithography approach.

## 2. Materials and Methods

Paraffin wax (semi-refined grade No. 58, melting point 58 °C, oil content <2%, conforming to GB/T 254-2022 [27]) was purchased from Fushun Chenyuechang New Material Products (Fushun, China). Cellulose paper (Whatman 1, thickness: 180 μm) was supplied by Cytiva (Uppsala, Sweden). Polydimethylsiloxane (PDMS, Sylgard 184) was purchased from Dow Corning (Midland, MI, USA). Purple food dye was from Fleur Couleur (Lianyungang, China). The silicon wafer was from Shunsheng Electronic Technology (Hangzhou, China). Photoresist (SU-8 2100) was obtained from Suzhou Yilan Microelectronics (Suzhou, China). The metal block was purchased from Dongguan Changping Hongxin Hardware Products (Dongguan, China). Glucose was purchased from Xilong Scientific (Shantou, China). Glucose oxidase (GOx) and horseradish peroxidase (HRP) were obtained from Sigma-Aldrich (St. Louis, MO, USA). The 3,3’-diaminobenzidine (DAB) chromogenic kit was purchased from Biosharp (Hefei, China). Urine samples are from healthy volunteers and spiked with glucose of different concentrations for experiments.

**Figure 1:**
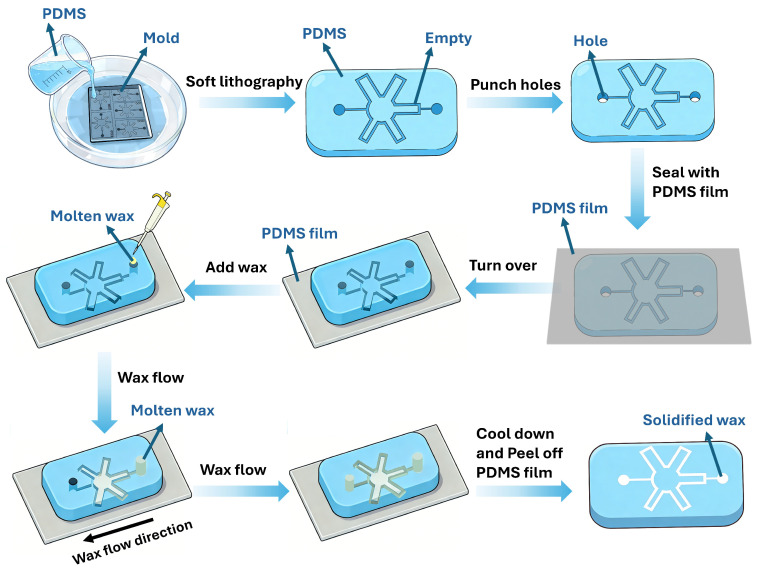
Procedures of preparing a PDMS chip with embedded wax. First, use soft lithography to cast PDMS onto the mold, replicating the microchannel pattern. Next, punch inlet and outlet holes, apply plasma treatment, and bond the patterned surface to a flat PDMS film. Then, introduce molten wax into the microchannel through one inlet. Once the wax fills the channel, allow it to cool and solidify. Finally, remove the PDMS film to obtain the wax-embedded structure.

For the fabrication of PDMS chips with embedded wax using soft lithography, the pattern of microfluidic channels was first designed by CAD software (AutoCAD 2025). The photomask was then printed, and the corresponding mold was fabricated via photolithography. During these processes, designed patterns are directly transferred to the photomask and subsequently to the SU-8 master mold. Therefore, the PDMS mold size is essentially identical to the design size, as soft lithography replication using PDMS faithfully reproduces the master mold features with high fidelity and negligible shrinkage. As shown in Figure 1, PDMS (base-to-curing agent ratio of 14:1) was cast onto the mold via soft lithography to replicate the microchannel structures. The higher base polymer content provides a more flexible texture, thereby producing PDMS that is better suited for wax pattern transfer [28,29]. The 14:1 ratio (base-to-curing agent) produces a PDMS that is more flexible than the conventional 10:1 formulation. During the thermal transfer step, this increased flexibility allows the PDMS chip to better conform to the microscopic roughness and fiber topography of the Whatman No. 1 paper surface. Improved conformal contact ensures that the wax, upon melting, is uniformly expelled from the PDMS microchannels into the paper along the intended pattern boundaries, minimizing lateral leakage and preserving resolution. Conversely, a stiffer PDMS (e.g., 10:1) would bridge across surface irregularities, creating gaps that allow molten wax to spread unpredictably beyond the designed channel edges. A softer PDMS (e.g., 20:1) can compromise the structural integrity of the microchannel features during the wax filling and thermal transfer steps. Thus, the 14:1 ratio balances sufficient flexibility for conformal contact with the paper surface while maintaining structural integrity to prevent channel collapse during wax introduction and solidification. Inlet and outlet holes were pre-punched on both sides of the PDMS chip. Before bonding, the PDMS chip was treated with oxygen plasma for hydrophilic treatment using a Harrick plasma cleaner (PDC-002, Harrick Plasma, Ithaca, NY, USA) with an RF power of 30 W for a duration of 60 s. After hydrophilic treatment, a PDMS film was placed on the microchannel side to form enclosed channels. Molten paraffin was then introduced into the enclosed channels through one of the pre-punched holes, allowing the molten wax to propagate throughout the microchannels under pressure-driven and capillary flow. Once the channels were completely filled, the device was allowed to cool, enabling the wax to solidify within the confined structures. Finally, the PDMS film was gently peeled off, yielding a PDMS chip with wax embedded in its microfluidic channels.

**Figure 2:**
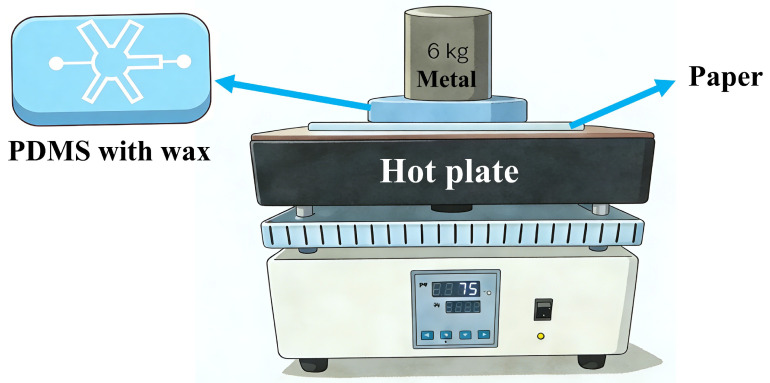
Diagram of the thermal transfer setup, comprising a hot plate (75 °C), a sheet of paper, a PDMS chip with embedded wax, and a 6.0 kg metal block providing uniform pressure.

Subsequently, thermal transfer was performed to transfer the wax patterns from the PDMS chip onto the paper substrate. As shown in Figure 2, a customized setup was used for the thermal transfer process. A sheet of cellulose paper was placed on a heating plate (DB-XAB, Lichen Instrument Technology, Shanghai, China) set to 75 °C, and the wax-embedded side of the PDMS chip was placed in contact with the paper. 75 °C was selected as the optimal balance. When the temperature exceeded 75 °C, the paraffin wax melted too rapidly, leading to excessive lateral flow and loss of pattern resolution. Below 75 °C, wax melting was incomplete or too slow, resulting in insufficient penetration into the paper and poor hydrophobic barrier formation. A 6 kg metal block was positioned on top to provide a stable, uniform load. Upon heating, the wax melted and penetrated into the paper along the predefined pattern under the influence of gravity and applied pressure. A 6 kg metal block was used to provide uniform pressure. Higher loads (>6 kg) caused irreversible deformation or crushing of the paper substrate. Lower loads (<6 kg) resulted in poor contact between the PDMS chip and the paper, leading to incomplete or uneven wax transfer and leakage during the thermal transfer step. After heating for 150 s, the PDMS chip and the metal block were removed, leaving behind wax-based hydrophobic barriers on the paper, thereby forming the microchannels. For the optimization of the heating time, the heating time was empirically determined to ensure complete melting of the paraffin wax, sufficient penetration into the paper pores, and no excessive lateral diffusion that would reduce channel resolution. A systematic optimization (varying heating time from 60 s to 300 s) was performed during method development. Too short a time (<120 s) led to incomplete wax transfer and poor barrier formation; too long (>180 s) caused spreading of wax beyond the designed patterns, decreasing resolution. The chosen 150 s balanced complete transfer with pattern fidelity. The functionality of the hydrophilic channels and hydrophobic barriers was then verified by introducing water or dye solutions. Notably, we specifically used Whatman No. 1 cellulose filter paper, which is the most commonly used substrate in μPADs fabrication. This choice ensures reproducibility and comparability with existing studies. The method’s parameters (75 °C, 150 s, 6 kg) were optimized specifically for this standard paper type. For other paper types, we believe that re-optimization would be required.

## 3. Results and Discussion

We designed a radial multi-channel microfluidic structure using CAD to investigate the minimum achievable channel width. The structure consists of seven rectangular microchannels radiating outward from a central circle, with designed widths of 1200 μm, 1300 μm, 1400 μm, 1500 μm, 1600 μm, 1700 μm, and 1800 μm. The structure was transferred onto a silicon wafer using photolithography. Subsequently, PDMS was cast onto the silicon mold to fabricate a PDMS chip with a channel width of 200 μm and a height of 84 μm. The channels of the PDMS chip were then filled with wax to obtain a PDMS chip with embedded wax. Finally, the pattern was transferred onto paper with a thickness of 180 μm via thermal transfer, thereby fabricating the channels on the paper. Then, water was introduced into the channels to assess the interface characteristics between hydrophilic and hydrophobic regions. Images were captured using a camera (Canon EOS RP, Tokyo, Japan), and the actual widths of the hydrophilic channels were measured using ImageJ Version 1.54s (https://imagej.net/ij/).

**Figure 3:**
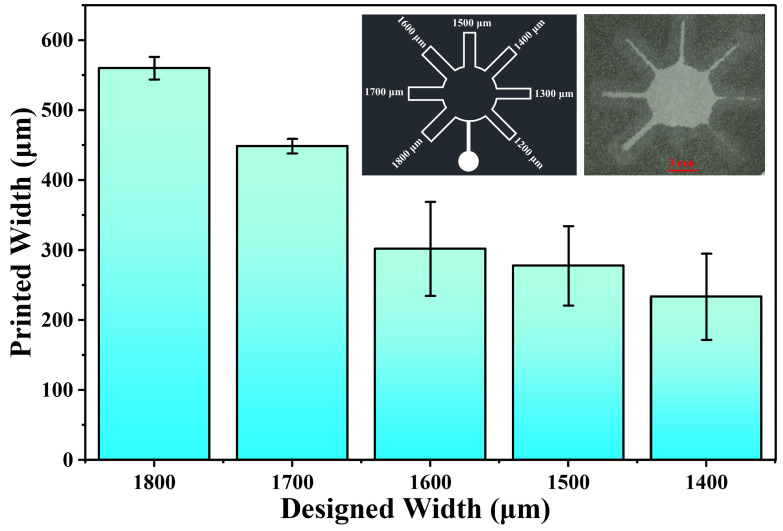
Printed widths corresponding to the designed microchannels of 1800 μm, 1700 μm, 1600 μm, 1500 μm, and 1400 μm. The left inset shows the CAD layout of the radial multi-channel structure, and the right inset displays the wax-printed pattern on paper fabricated using wax soft lithography. Each experiment is repeated three times.

Figure 3 shows the printed channel widths corresponding to the designed channel widths. As shown in the bar chart, there is a clear downward trend in the printed channel width as the designed width decreases from 1800 μm to 1400 μm. For a design width of 1400 μm, the minimum achievable width of the hydrophilic channels was 234 ± 62 μm, demonstrating higher resolution and patterning precision than the work by Su et al. [26] (detailed comparison can be found in Table S1 in Supplementary Information). Among all measured conditions, the 1400 μm design yielded the smallest average printed channel width (234 μm) but the largest relative variability (26% CV). This level of variability is higher than that reported in several representative μPAD fabrication methods. For example, Dungchai et al. achieved a minimum hydrophilic channel width of 650 ± 71 μm (11% CV) using wax screen-printing [30], while Su et al. reported 654 ± 75 μm (12% CV) [26]. Carrilho et al. further demonstrated 561 ± 45 μm (8%) using a commercially available printer [15]. This increased variability is attributed to the fact that this design approaches the practical resolution limit of the wax soft lithography method on Whatman No. 1 paper. At such narrow channel widths, minor variations in paper fiber orientation, local porosity, wax penetration uniformity, and slight inconsistencies in PDMS-paper contact pressure during thermal transfer can lead to proportionally larger fluctuations in the final hydrophilic channel width. In contrast, wider channels (≥1500 μm design width) are more tolerant to these factors, resulting in smaller relative errors. This observation underscores the trade-off between resolution and reproducibility near the fabrication limit. However, when attempting to fabricate channels with designed widths of 1300 μm and 1200 μm, the resulting structures were excessively narrow to support water flow. As a result, the printed channel widths could not be quantified. The minimum achievable channel width is likely limited by the balance between wax spreading during thermal transfer and the intrinsic pore size and fiber distribution of the cellulose paper (Whatman No. 1). Below a certain design width, the wax from adjacent hydrophobic regions may merge, or the hydrophilic channel may become too narrow to maintain a continuous wicking path. By comparison with Su et al. and Tenda et al. (228 ± 30 μm via wax printing) [16,26], our method achieves resolution comparable to high-end wax printing but with simpler equipment and without the need for a dedicated wax printer. The 234 μm resolution is sufficient for many point-of-care applications, as demonstrated by our following glucose detection. The resolution improvement over the laser-cut-based method (Su et al. [26]) primarily originates from the use of a photolithographically defined SU-8 master mold. Photolithography produces vertical sidewalls, low surface roughness, and precise critical dimensions, which are faithfully replicated into the PDMS mold. During thermal transfer, these sharp mold features confine molten wax with minimal lateral spreading. In contrast, laser-cut masters exhibit kerf-induced roughness and tapered sidewalls, leading to irregular wax distribution and lower resolution.

**Figure 4:**
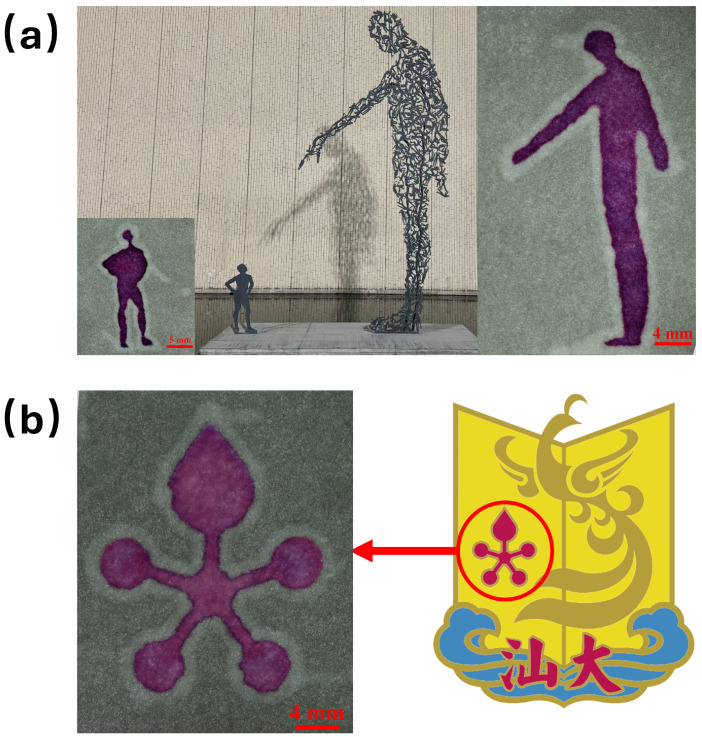
Pattern fabrication on paper using wax soft lithography. (**a**) Wax patterns replicating the “Big Man–Little Man” sculpture at Shantou University. (**b**) Wax pattern based on the Flame Tree flower from the emblem of Shantou University. The two Chinese characters in the emblem are the Chinese abbreviation for Shantou University.

We also designed molds with various patterns using CAD and fabricated corresponding wax patterns on paper via wax soft lithography. Figure 4 displays the pattern structures obtained from different designs. Figure 4a shows the outline of the iconic “Big Man–Little Man” sculpture at Shantou University. Figure 4b displays a pattern designed based on the Flame Tree flower from Shantou University’s emblem. After introducing a purple dye solution into the patterned regions, the shapes were clearly visualized with no dye leakage, indicating that the wax boundaries effectively confined the liquid and functioned as reliable hydrophobic barriers.

**Figure 5:**
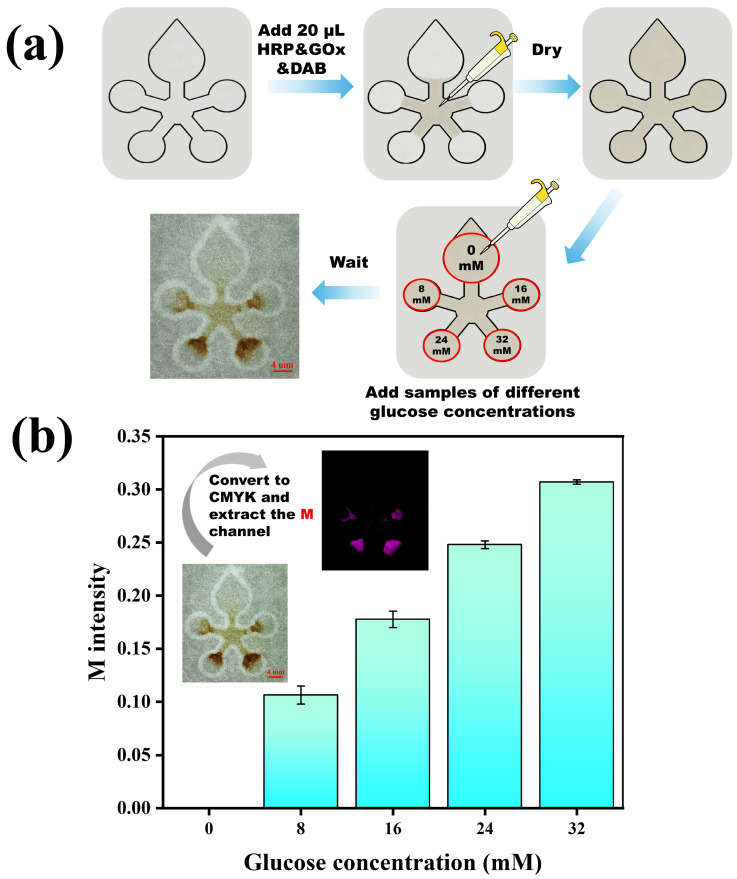
Glucose detection in urine on μPADs fabricated by wax soft lithography. (**a**) Schematic of the detection procedure: 20 μL of immunoreagents (GOx, HRP, and DAB mixture) was added to the center. After drying, spiked urine samples of different glucose concentrations were added to designated zones. (**b**) Detection results for urine samples of different glucose concentrations, including the original images, the M-channel images extracted from the CMYK color space, and bar charts based on M-channel intensity. Each experiment is repeated three times.

As a proof of concept for using such μPADs in point-of-care diagnostics, we employed the fabricated Flame Tree flower pattern to detect glucose in spiked urine samples. As shown in Figure 5a, we first added 20 μL of a mixed solution containing horseradish peroxidase (HRP), glucose oxidase (GOx), and 3,3’-diaminobenzidine (DAB) to the center, allowing it to fill the microchannels and dry. We then added urine samples of different glucose concentrations to designated detection zones and let them react for 10 min before taking photographs. We extracted the Magenta channel values using the RGB to CMYK conversion function in ImageJ for quantitative analysis. As shown in Figure 5b, the M color intensity increased with glucose concentration. The preliminary results on the μPADs show a consistent trend and acceptable reproducibility, suggesting potential for further quantitative biosensor development. Normal urine glucose concentration is typically below 1.8 mM, while diabetic glucosuria often exceeds 2.8 mM. Our tested range (0–32 mM) covers and extends beyond this clinically relevant window, and the M-channel intensity increases monotonically within the 0–15 mM range, indicating potential utility for diabetes screening and monitoring. It should be noted that this glucose experiment serves as a proof-of-concept demonstration of the fabricated μPADs’ functionality, not a fully validated analytical assay. Comprehensive figures of merit (e.g., calibration curve with R^2^, LOD, LOQ, selectivity against common interferents, and spike-and-recovery) are important for clinical translation, and need further validation before practical applications in the near future.

For the cost of this method, although the fabrication of the master mold requires photolithography equipment and materials (e.g., SU-8, silicon wafer), which incur moderate upfront costs, the subsequent wax soft lithography process allows for low-cost, high-resolution production of μPADs per device, especially when replicating from a single PDMS mold. Researchers without access to photolithography may also obtain ready-made masters via collaboration or commercial services.

## 4. Conclusions

In summary, we developed a novel fabrication method for μPADs by integrating wax printing with soft lithography, termed wax soft lithography. We systematically evaluated the minimum achievable channel width, demonstrating that this method can produce microchannels with a high resolution of 234 ± 62 μm. We also successfully demonstrated its capability for the rapid construction of patterns with complex shapes. As a proof of concept, we used the μPADs to detect glucose in urine samples of varying concentrations, confirming their stable and reliable performance. This approach substantially lowers the barrier to producing high-precision μPADs in resource-limited settings and shows strong potential for future development toward POCT applications, pending full analytical validation.

## Data Availability

The original contributions presented in the study are included in the article; further inquiries are available per request addressed to guoweijin@stu.edu.cn.

## Supplementary Materials

The following supporting information can be downloaded at: https://www.mdpi.com/article/10.3390/mi17050512/s1, Table S1: Difference between our work and the work by Su et al.; Table S2: Comparison of Different Wax Patterning Methods for Paper-Based Devices; Figure S1: Measurement of microchannel width; Figure S2: The time–distance curves for water capillary flow in each of the 7 channels; Figure S3: Statistical analysis of M intensity at various glucose concentrations (0, 8, 16, 24, 32 mM). *T*-test was performed to evaluate statistical significance between adjacent concentration groups. Asterisks denote the level of significance: ****p* < 0.001. Each experiment is repeated three times.; Figure S4: Cross-sectional SEM characterization of wax-impregnated Whatman No. 1 filter paper: (a) Overview of the paper cross-section. (b) Unwaxed region showing the native porous structure. (c) Wax-impregnated region with full penetration.
