# Supplementary material for: Fabrication of Paper Microfluidic Chips via Wax Soft Lithography
Source: Micromachines (Basel). 2026 Apr 23;17(5):512. doi: 10.3390/mi17050512 (PMC13209656; doi:10.3390/mi17050512)
Supplement: Supplementary file 1 [file micromachines-17-00512-s001.zip › micromachines-4238611-supplementary.pdf]

Supplementary Materials

Fabrication of Paper Microfluidic Chips via Wax Soft Lithography

Xinyi Chen, Jie Zhou, Jiahua Zhong, Zitong Ye, Qinghao He, Hao Chen and Weijin Guo*

Department of Biomedical Engineering, Shantou University, 243 Daxue Road, Shantou, 515063, Guangdong, China.

*Corresponding author. E-mail(s): guoweijin@stu.edu.cn

Comparison with previous work

Both our work and the work by Su et al. (Ref. 26: Distinctive Prototyping of Paper-Based Microfluidic Devices via Capillary-Driven Wax Patterning. ACS omega, 2025, 10, 50046–50054) utilize a combination of wax and PDMS for paper-based microfluidic device fabrication. However, there are several critical differences in methodology, resolution, and overall innovation, which are summarized below in Table S1.

Table S1. Difference between our work and the work by Su et al.

Aspect	**This Work (Wax Soft Lithography)**	**Ref. [26] (Su et al.)**
**Master Mold Fabrication**	**Photolithography** (SU-8 on silicon wafer) – high precision, standard microfabrication.	**Laser cutting of self-adhesive paper** – lower cost but lower resolution and rougher sidewalls (trapezoidal channels as shown in their SEM images).
**Wax Transfer Mechanism**	Wax is filled into **PDMS channels sealed with a PDMS film**, then the entire PDMS chip (with embedded wax) is placed directly onto paper for thermal transfer.	Wax is patterned onto a **glass substrate** first (forming a wax stamp on glass), then the glass-wax assembly is heated and pressed onto paper.
**Minimum Channel Width Achieved**	**234 ± 62 μm** (for a designed width of 1400 μm).	654 ± 75 μm.
**Channel Geometry & Resolution**	Demonstrated complex patterns with clear hydrophobic barriers and no dye leakage.	Demonstrated basic patterns (circular, bow-shaped, radial channels) but with less emphasis on sub-millimeter precision in the final paper device.
**Application Demonstration**	**Glucose detection in urine** – quantitative analysis using M-channel intensity from CMYK conversion, with a clear concentration-response relationship.	**Total amino acid detection in tea** – colorimetric assay using ninhydrin, with mean gray intensity analysis.
**PDMS Ratio**	**14:1** (base:curing agent) – softer PDMS for better conformal contact and wax transfer.	Standard 10:1 ratio (implied from Sylgard 184 kit, though not explicitly stated).

The primary innovation of our work lies in integrating photolithography-defined master molds with a PDMS-film-sealed wax-filling process, followed by direct thermal transfer from PDMS to paper. This differs fundamentally from Su et al., where wax is first transferred to an intermediate glass substrate. Our approach:

• Eliminates the intermediate glass stamping step, reducing potential alignment issues and fabrication complexity.

• Uses photolithography (instead of laser-cut paper masters) to achieve smoother channel walls and more reproducible microscale features.

• Achieves a functional hydrophilic channel width of 234 ± 62 μm directly on paper, which is competitive with wax printing and better than many low-cost methods, while avoiding the need for expensive wax printers or photolithography directly on paper.

The main contributions of our work relative to Su et al. and the broader field are:

• First demonstration of "wax soft lithography" that combines the fidelity of SU-8 photolithography with the simplicity of wax patterning on paper, using a PDMS-film-sealed chip.

• Systematic quantification of the minimum achievable hydrophilic channel width (234 ± 62 μm) under defined design parameters, including explicit reporting of failure conditions (designed widths <1300 μm).

• High-resolution complex pattern fabrication (e.g., university sculpture and emblem) that validates the method's capability for intricate, application-specific designs.

• Practical validation in a clinically relevant POCT application – glucose detection in urine – with quantitative image analysis (CMYK M-channel), demonstrating reproducibility and potential for resource-limited settings.

• Lower equipment barrier compared to photolithography-on-paper or high-end wax printing, while achieving comparable or better resolution than many laser-cutting-based approaches.

In summary, while Su et al. presents a capillary-driven wax patterning method using laser-cut masters and glass intermediates, our work offers a higher-resolution, more reproducible, and directly transferable PDMS-to-paper approach using photolithography-defined masters. The 234±62 μm resolution, combined with successful glucose detection in urine and complex pattern fabrication, represents a clear advancement and a practical contribution to low-cost, high-resolution paper-based microfluidics for POCT.

Comparative analysis of patterning techniques

Table S2 provides a comparison of four methods—photolithography (Ref. 18: Klasner et al., Paper-Based Microfluidic Devices for Analysis of Clinically Relevant Analytes Present in Urine and Saliva. Analytical and Bioanalytical Chemistry, 2010, 397, 1821–1829), wax printing (Ref. 16: Tenda et al., High-Resolution Microfluidic Paper-Based Analytical Devices for Sub-Microliter Sample Analysis. Micromachines, 2016, 7, 80), capillary-driven wax patterning (Ref. 26: Su et al., Distinctive Prototyping of Paper-Based Microfluidic Devices via Capillary-Driven Wax Patterning. ACS Omega, 2025, 10, 50046–50054), and the proposed wax soft lithography—across four key parameters: resolution, equipment cost, scalability, and patterning principle. This comparison contextualizes the performance of the proposed method against existing techniques. As shown, the proposed method (234 ± 62 μm) achieves a resolution comparable to that of wax printing (228 ± 30 μm) and substantially better than that of capillary-driven wax patterning (654 ± 75 μm), although it does not surpass photolithography (~90 μm). The equipment cost for the proposed method is classified as middle, similar to the wax printing and capillary-driven approaches.

Table S2. Comparison of Different Wax Patterning Methods for Paper-Based Devices

Method	Photolithography	Wax Printer	Capillary-Driven Wax Patterning	Wax Soft Lithography
Resolution	~90 μM	228 ± 30 μM	654 ± 75 μm	234 ± 62 μM
Equipment cost	High (Photolithography machine)	Middle (Wax printer, discontinued)	Middle (Laser cutter)	Middle (Photolithography machine)
Scalability	Poor	Good	Good	Good
Patterning Principle	UV photolithography cross-linked polymers form hydrophobic barriers	Double-sided wax printing and hot lamination to form hydrophobic barriers	Wax pre-patterned on glass as a stamp is transferred via heating and pressing	Wax embedded in PDMS chips is thermally transferred directly onto paper

Channel width definition and measurement

Figure S1 illustrates the method for measuring the microchannel width at the channel–hydrophobic barrier interface. The channel width is defined as the distance between the two edges of the hydrophobic wax barrier, measured perpendicular to the channel axis.

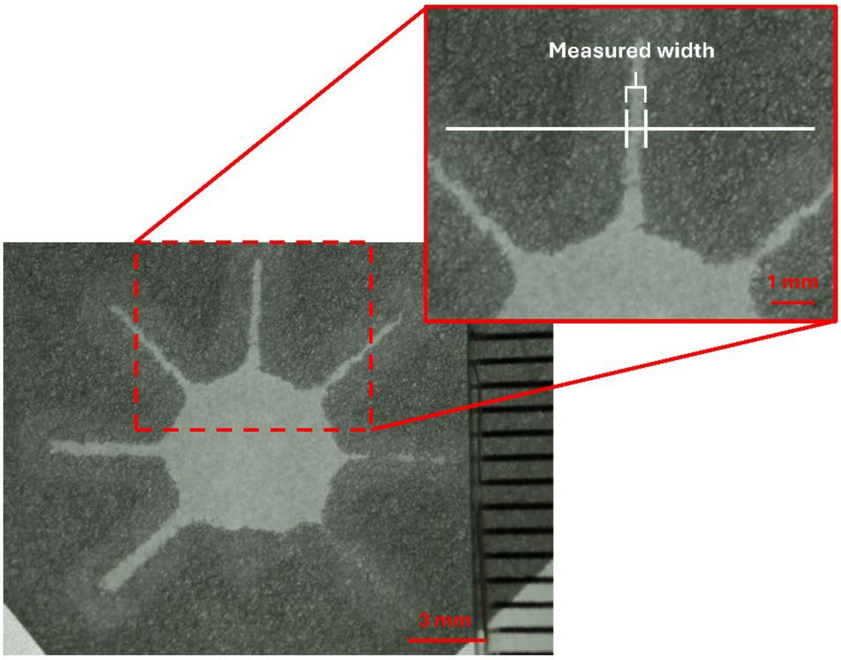

Figure S1. Measurement of microchannel width

Capillary flow characterization of the multi-channel microfluidic device

Capillary flow tests were conducted on the 7-channel radial microfluidic chip by introducing fluid into the central reservoir. As shown in Figure S2, the time–distance curves of all channels show highly consistent flow behavior, demonstrating good flow uniformity and repeatability, with all branches filling almost simultaneously.

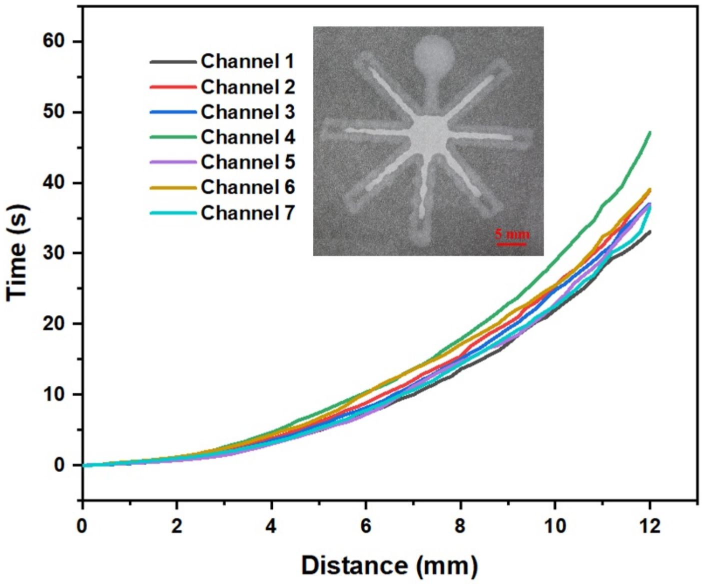

Figure S2. The time–distance curves for water capillary flow in each of the 7 channels

Statistical significance of glucose concentration effects

To rigorously validate the concentration-dependent response of the M intensity signal to glucose concentration, we have performed t-test to assess the statistical significance between all adjacent concentration groups. Figure S3 shows the results of t-test.

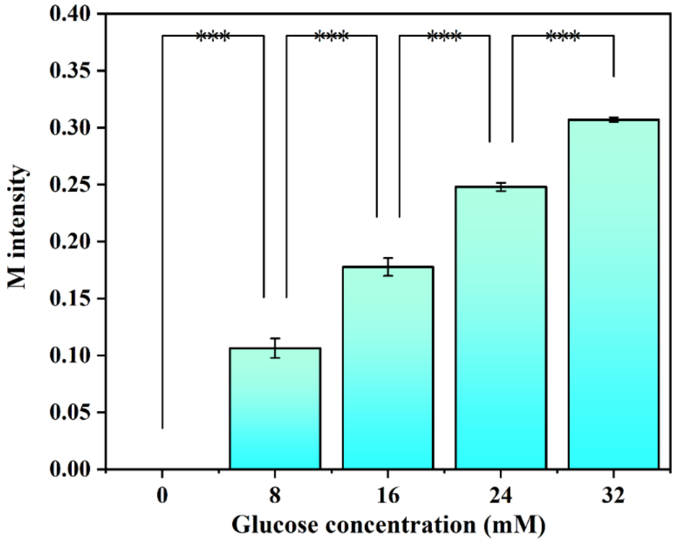

Figure S3. Statistical analysis of M intensity at various glucose concentrations (0, 8, 16, 24, 32 mM). T-test was performed to evaluate statistical significance between adjacent concentration groups. Asterisks denote the level of significance: ***p < 0.001. Each experiment is repeated three times.

Cross-sectional SEM of wax-impregnated paper

Figure S4 shows cross-sectional images of the wax-patterned filter paper. Figure S4(b) displays the unwaxed region with a porous fibrous network, while Figure S4(c) presents the wax-impregnated region, where wax fully penetrates the paper to form a dense, continuous barrier without gaps.

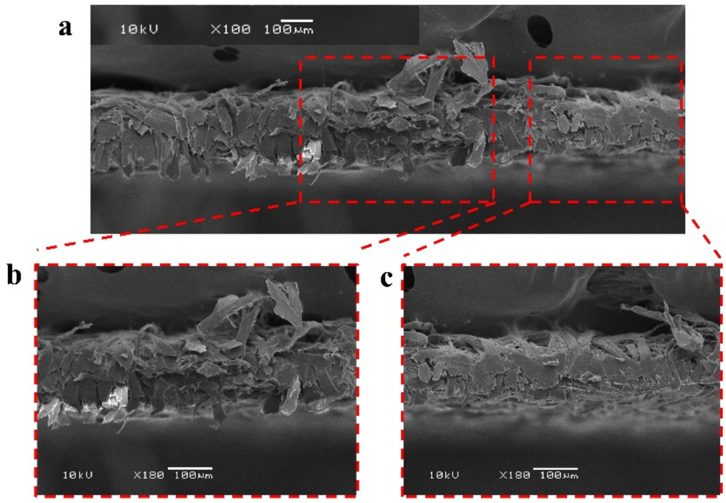

Figure S4. Cross-sectional SEM characterization of wax-impregnated Whatman No. 1 filter paper: (a) Overview of the paper cross-section. (b) Unwaxed region showing the native porous structure. (c) Wax-impregnated region with full penetration.
